# Comparison of veterinary health services expectations and perceptions between oncologic pet owners, non-oncologic pet owners and veterinary staff using the SERVQUAL methodology

**DOI:** 10.14202/vetworld.2016.1275-1281

**Published:** 2016-11-18

**Authors:** Hugo Gregório, Patricia Santos, Isabel Pires, Justina Prada, Felisbina Luísa Queiroga

**Affiliations:** 1 Veterinary Hospital Centre, Rua Manuel Pinto de Azevedo 118, 4100-320 Porto, Portugal; 2 Department of Veterinary Sciences, University of Trás-os-Montes and Alto Douro, 5001-801 Vila Real, Portugal; 3 Animal and Veterinary Research Centre (CECAV), University of Trás-os-Montes and Alto Douro, 5001-801 Vila Real, Portugal; 4 Center for Research and Technology of Agro-Environment and Biological Sciences (CITAB), University of Trás-os-Montes and Alto Douro, 5001-801 Vila Real, Portugal

**Keywords:** expectations, oncology, perceptions, SERVQUAL, veterinary

## Abstract

**Aim:**

Client satisfaction gained great importance in health care as a measurement of service quality. One of the most popular methods to evaluate client satisfaction is the SERVQUAL inquiry which measures service quality by evaluating client expectations and services towards a service in five dimensions: Tangibles, Empathy, Assurance, Reliability and Responsiveness.

**Materials and Methods:**

In order to evaluate if owners of pets with cancer constitute a distinctive group from the general pet owner population and if these differences were perceived by the hospital staff we applied a SERVQUAL questionnaire to 51 owners of pet with cancer, 68 owners from the general pet population and 14 staff members.

**Results:**

Owners of oncologic pets had different expectations of an ideal service granting importance to Assurance questions (6.75 vs 6.5, p= 0.045) while showing unmet needs in Reliability and Empathy dimensions. Veterinarians failed to understand these specificities and over evaluated characteristics of Tangible dimension (6.75 vs 6.25, p=0.027).

**Conclusion:**

Owners of pet with cancer seem to constitute a specific subpopulation with special needs and veterinary staff should invest resources towards Assurance instead of privileging tangible aspects of veterinary services. By aligning professionals expectations with those of pet owners veterinarians can achieve better client satisfaction, improved compliance and stronger doctor-owner relationships.

## Introduction

For a while now, the quality of service provided to customers became a major concern in many different service industries including health care and veterinary care. Recent studies identified several challenges to the veterinary industry including differences in perception of the importance of veterinary services between veterinarians and pet owners [1-4].

To address service quality, a group of researchers developed, and later refined, a functional tool named SERVQUAL that aimed to measure the quality of a service or at least what customers perceived as quality [5-7]. The researchers developed the scale with the knowledge that evaluating the quality of a service is much more complex than evaluating a product. They considered that the universe of SERVQUAL was composed by five different dimensions common to every business service industry [5]. Those five dimensions included tangibles (physical facilities, equipment and appearance of personnel), reliability (the ability to perform the promised service in a dependable and accurate fashion), responsiveness (the willingness to help customers and provide prompt service), assurance (the knowledge and courtesy of employees and their ability to inspire trust and confidence), and empathy (the individualized attention the firm provides to its customers) [6,7].

At SERVQUAL’s final form, after the lengthy refinement process, the tool included a pair of 22 items the first of which focused on the participant’s expectations and the second on the participant’s perceptions about a given service [7].

SERVQUAL is contextualized within what is considered functional quality (“caring”) of service, as opposed to technical quality (“curing”) which is based on the technical precision of procedures and involves an objective aspect of an event whereas functional quality implicates a subjective response from clients to what is presented to them [6]. The manner in which the service is delivered to the customer (functional quality) is what is considered to be one of the most valuable determinants in whether a client intends to purchase a service or not [5,6]. Service clients (pet owners) are unable to evaluate the technical quality of a given service with the technical expertise of a veterinarian (e.g., the quality of surgery performed or whether medical protocols are up to date). Even though technical quality can be measured, by the comparison of success rates of procedures for example, or even by the comparison of overall survival rates in particular diseases among different hospitals, this type of information is not usually available to medical patients or pet owners. As so, pet owners rely on evaluating and quantifying the functional component of SERVQUAL the one that customers are aware of and can actually give their feedback [8,9]. This includes aspects such as the promptness of the service, the physical quality of the facilities, and sympathy of the staff and makes it possible for a pet owner to be more satisfied with a lesser technical quality service.

The SERVQUAL instrument is used to quantify this functional component in which the measurement of service quality (Q) is achieved by subtracting the client’s perceptions (P) to his/her expectations (E): Q=P−E [6]. If the gap score is positive, it implies that the customer’s expectations were exceeded, synonym of a service with excellent quality and high levels of satisfaction. However, a negative score indicates the opposite. Expectations can be classified as predictions made by consumers about what is likely to happen during a transaction, whereas perception is the actual recognized experience. This instrument is used across a wide variety of business services with minor alterations to the original design which included several health-care services such as clinics and hospitals [8-10], nursing care [11], dental care [12], and clinical laboratories [13]. In veterinary medicine, few studies addressed pet owner expectations and were limited to financial, information and communication aspects, and none used the SERVQUAL methodology [14-17].

Regarding owners of pets with cancer, little to no information has been collected about their expectations in veterinary health-care services. Human cancer patients [18] and care givers of people with cancer experience severe distress [19] and appear to show specific needs when compared with caregivers of people with other chronic diseases [20,21] and that might also be true in owners of cancer-bearing pets. Human patients with cancer also showed different expectations from their medical team [22]. The uncertainty of the future and well-being of a dear companion can cause a great amount of stress and grief to many individuals that consider their pets as integral members of the family [23,24]. Moreover, unmet communication expectations of pet owners can lead to deterioration of owner-veterinarian relation leading to poorer compliances and health-care results [25]. As cancer becomes more prevalent among veterinary patients and new treatment modalities become available leading to increasing overall survival, veterinarians will be challenged to deal more often with cancer-bearing animal owners. Several studies in human medicine have addressed this issue. Several gaps have been detected and usually physicians were not aware of these gaps and tended to overestimate their performance scores and underestimate patients expectations leading to patient dissatisfaction [26,27]. Staff members tended to underestimate reliability, assurance, responsiveness, and empathy and to overvalue tangibles when compared with patients [26,27].

We hypothesize that owners of pets with cancer might constitute a specific population among general pet owner population with special needs and expectations that might not be addressed by veterinary professionals.

The final objective of this study is to analyze and compare expectations, perceptions, and general service satisfaction between owners of pets treated for cancer and owners of pets presented for non-cancer disease to clarify if the former constitutes a specific group with special needs and expectations providing veterinarians information and tools to meet this population needs and ambitions. By identifying specific gaps between different groups of pet owners and veterinary professionals, specific recommendations and alterations could be performed better adequate veterinary services to client needs.

## Materials and Methods

### Ethical approval

All procedures were carried out according to the international practices for animal use and care under the control of an internal committee of the Porto Veterinary Hospital Centre as complying with the Portuguese legislation for the protection of animals (Law no. 92/1995, from September the 12^th^). Informed consent was obtained from all pets owners.

### Study design

The study consisted of modified SERVQUAL questionnaires delivered to pet owners and veterinary staff of a private veterinary hospital located in Porto, Portugal in a period ranging from 1 of January of 2013 to 28 of February of 2015.

### Study participants

The sample consisted in oncologic pet owners (oncologic group - OG) and non-oncologic pet owners (general group - GG), all of which were attended by the same veterinary professional, the first author. The last group was composed by customers that brought their pets in for a variety of chronic conditions other than oncologic disease but that required hospitalization or regular appointments. First appointment or routine appointments were excluded as were referred clients.

Owners were eligible for the OG of this study if their pet had any type of cancer and/or were receiving any form or a combination of ongoing treatment (surgery or chemotherapy); prior knowledge of a cancer diagnosis was also required. Clients with pets ongoing chemotherapy treatment that had long duration or customers that visited the installations with their pets more than once during the extent of the study were asked to spare information only once at the beginning of treatment. Owners of pets only subjected to surgery were asked to answer the questionnaire in the recheck appointment just after surgery.

Potential pet owner’s participants were approached by an element of the hospital’s staff, usually by one of the studys’ authors during the pet’s consultation regarding its health condition. Clients were explained the general purpose of the study. Those willing to participate in the study received a questionnaire to fill-up. Regarding the GG, owners were selected if their pets presented a non-oncologic chronic disease and did not present a history of cancer in the last 5 years.

The staff of the veterinary hospital, where the study was conducted, was also invited to fill up the first section of the questionnaire so that the veterinary professionals and owners expectations could be compared.

### Questionnaire design

Each owner was asked to complete a modified SERVQUAL survey. Although most of the questionnaires were handed out by the authors of this study, a few were delivered by the desk clerk or by a nurse on duty so that the customer would have the opportunity to fill-up the survey while they were waiting at the reception for a consultation with the hospital’s doctor. The SERVQUAL questionnaire included 22 pairs of items. The first pair was dedicated to the participant’s expectations while the second pair acknowledged the customer’s perspectives regarding the service that had just been provided. The five SERVQUAL dimensions were distributed by the following manner: Tangibles (item 1-4), empathy (item 5-9), responsiveness (item 10-13), assurance (item 14-17), and reliability (item 18-22).

### Data analysis

The classification of each item by the participant was made by selecting the most appropriate score using a 7-point Likert scale anchored on one end (1) by “completely disagree” and on the other end (7) by “completely agree,” with no verbal labels from 2 to 6. The standard SERVQUAL survey had to be adapted with terms that more accurately described a veterinary hospital’s environment and that were more fitting to the study. Terms such as “veterinary hospital,” “veterinary hospital employees,” and “pet” were included in the study. Other sentences were slightly altered to maintain the coherence of the whole statement. The final version of the survey is included in the “supplementary data” section of this study.

The attainment of results for both client expectations and perceptions was achieved by calculating the median scores for each dimensions and overall and comparing them between both client sample groups. The SERVQUAL satisfaction measurement was obtained by application of the main formula: Q=P−E.

The gaps among client expectations and the veterinary professionals’ notions of what their clients expect were obtained by calculating the median scores for all five SERVQUAL dimensions and comparing them between the two client groups and the group which contained the veterinary doctors and nurses.

### Statistical analysis

Data analysis was performed using SPSS 18 (IBM Corporation, USA). Differences in scores between groups were compared using Kruskal–Wallis and Mann–Whitney tests.

## Results

Data from 51 OG owners, 68 GG owners, and 14 veterinary staff (8 doctors and 6 nurses) were retrieved.

### Owner expectations

Client expectations were compared between GG and OG by calculating the median score among the distinct dimensions. Total expectations score was higher in OG although not statistically significant (145 vs. 142.4; p=0.251). For the OG, the highest median score can be attributed to the empathy dimension (6.80), followed by the assurance and responsiveness dimensions, both with a median score of 6.75. Reliability achieved a median score of 6.50 while tangibles obtained the lowest median score for the OG, with 6.25 (Table-1). As for the GG, the highest median score was also assigned to the empathy dimension (6.60). Tangibles, reliability and assurance dimensions all obtained a median score of 6.50. The poorest outcome was related to the responsiveness dimension, with a score of 6.25. The distribution of scores was compared among both groups regarding each dimension individually. The OG granted higher expectations to the assurance dimension in comparison with the GG (p=0.045). The difference was statistically significant (significant at p<0.05). Furthermore, the same group tended to overvalue tangibles (p=0.055) and responsiveness (p=0.056) dimensions. The difference was not statistically significant for the reliability (p=0.171) and empathy (p=0.132) dimensions.

**Table 1:** Summarized information concerning the results obtained for client expectations.

Service dimensions	OG	GG	p
Total	145.00 (137.00; 151.00)	142.50 (134.25; 149.00)	0.251
Tangibles	6.25 (5.50; 7.00)	6.50 (6.06; 7.00)	0.055
Reliability	6.60 (6.20; 7.00)	6.50 (6.05; 6.80)	0.132
Responsiveness	6.75 (6.00; 7.00)	6.25 (5.75; 6.75)	0.056
Assurance	6.75 (6.25; 7.00)	6.50 (6.25; 6.75)	0.045
Empathy	6.80 (6.40; 7.00)	6.60 (6.20; 6.95)	0.171

Results presented as median, 25% percentile and 75% percentile. OG=Oncologic group, GG=General group

### Owner perceptions

Client perceptions were also compared among both sample groups (Table-2). Overall perceptions of participants of the OG and the GG did not differ (150 vs. 146; p=0.206).

**Table 2:** Summarized information concerning the results obtained for client perceptions.

Service dimensions	OG	GG	p
Total	150.00 (142.00; 154.00)	146.00 (137.75; 154.00)	0.206
Tangibles	6.75 (6.25; 7.00)	6.75 (6.25; 7.00)	0.962
Reliability	6.80 (6.20; 7.00)	6.60 (6.20; 7.00)	0.743
Responsiveness	7.00 (6.25; 7.00)	6.63 (6.00; 6.75)	0.042
Assurance	7.00 (6.50; 7.00)	6.75 (6.25; 6.75)	0.005
Empathy	6.80 (6.40; 7.00)	6.80 (6.40; 7.00)	0.531

Results presented as median, 25% percentile and 75% percentile. OG=Oncologic group, GG=General group

In the OG, responsiveness and assurance were the dimensions with the highest median score, 7.00, followed by empathy and reliability, both with a score of 6.80. Finally, the tangibles dimension obtained a median score of 6.75 (Table-2). In the GG, the highest median score can be attributed to the empathy dimension, with 6.80, followed by the tangibles and assurance dimensions, both with a score of 6.75. Responsiveness obtained a median score of 6.63 for client perceptions, whereas reliability presented the lowest score, with 6.60 (Table-2). Client perception scores were also compared between GG and OG, and a significant difference was obtained for both responsiveness and assurance dimensions, with a p value of 0.042 and 0.005, respectively.

### SERVQUAL Score

SERVQUAL score was calculated by applying for both test groups the formula: Q=P−E. These calculations were made in regard to the mean scores of each dimension and the global mean score.

Overall, SERVQUAL score was slightly higher in the GG (59.0) when comparing with the OG (56.8), but this difference was not statistically significant (p=0.729).

**Figure-1:**
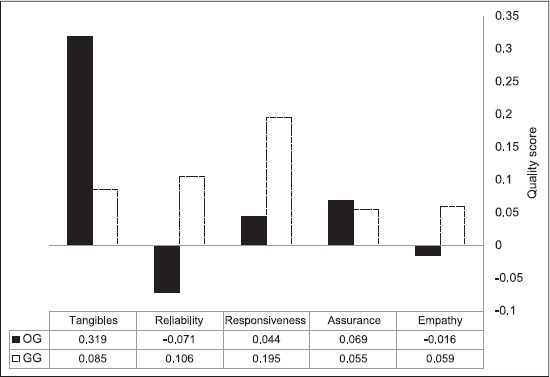
Service quality scores per dimension.

The OG presented some unmet expectations: The reliability and empathy dimensions presented negative values for Q (SERVQUAL), with scores of −0.071 and −0.016, respectively. The remaining dimensions obtained positive results with assurance presenting a score of 0.069 and responsiveness with 0.044. Tangibles achieved the highest SERVQUAL score for the OG, with 0.319 (Figure-1). The results are far more satisfying in the GG, where we can find positive scores for every dimension, meaning that the expectations of these clients were not only met, but exceeded. The dimension with the highest SERVQUAL score is responsiveness, with 0.195, followed by reliability, with 0.106 and tangibles, with a score of 0.085. The empathy and assurance dimensions also obtained positive scores, with 0.59 and 0.55, respectively.

### Veterinary professionals and owners expectations

**Figure-2:**
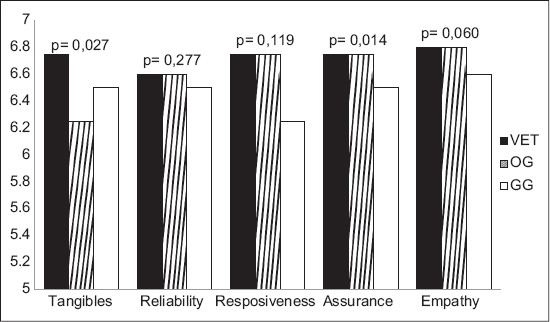
Expectation scores by dimension and group.

Veterinary professionals exhibited higher expectation throughout all five dimensions with exception of empathy (Figure-2). The highest median score was attributed to the empathy dimension, with 6.80, followed by the tangibles, responsiveness and assurance dimensions, all with a score of 6.75. Finally, reliability appears in the last place, with 6.60 (Table-3 and Figure-2).

**Table 3:** Summarized information regarding median expectations scores per dimension for all sample groups.

Service dimensions	VET median score	OG median score	GG median score
Tangibles	6.75	6.25	6.5
Reliability	6.6	6.6	6.5
Responsiveness	6.75	6.75	6.25
Assurance	6.75	6.75	6.5
Empathy	6.8	6.8	6.6

OG=Oncologic group, GG=General group

A Kruskal–Wallis test was performed comparing data from the OG expectations, the GG expectations and the veterinary professional’s expectations. Differences of distribution were noticeable among the tangibles dimension (p=0.027) and the assurance dimension (p=0.014). Consecutively, individual Mann–Whitney tests were performed between the veterinary professionals group and the GG, as well as among the veterinary professionals group and the OG for these two dimensions. Results exhibited statistical significant distribution differences for the assurance dimension (p=0.007), regarding the test that compared professionals to members of the GG. As for the test relating OG and veterinary professionals, statistically significant differences were noted only for the tangibles dimension (p=0.019).

## Discussion

Relatives of people suffering from cancer experience extreme stress and anguish making them a subpopulation with special needs and expectations and at risk, for example, for depression [28]. The increasing strength of the human-animal bond contributes to the fact that many people considered their pets family members [29] making them candidates for experiencing the same needs as people with diseased relatives [30,31]. Meanwhile, the increasing prevalence of cancer in pets followed by the development of new treatment modalities contributes to an increasing caseload of veterinary cancer patients among veterinary care. Veterinary professionals need to deal with owner anxiety regarding their pets who, due to the chronicity of their disease, need to visit and communicate often with veterinary professionals putting a strain in doctor-owner relationship. Hence, it is of paramount importance that veterinary professionals know and meet their client’s expectations and needs to increase owner satisfaction and decrease doctor frustration and anxiety associated with doctor-owner relationship aggravation and to acknowledge where improvements are needed from the owner perspective.

This work confirmed the presence of four gaps identified by the SERVQUAL methodology applied to veterinary services: Different particular expectations between expectations of GG and OG (Gap 1), different perception of veterinary services between GG and OG (Gap 2), different service satisfaction among GG and OG (Gap 3), and unaligned expectations of what an excellent veterinary service constitutes for veterinary professionals and veterinary clients (Gap 4).

There seems to be no significant difference in overall expectations between OG and GG meaning that generally speaking owners of pets with cancer are not more demanding than owners from the general population. The first gap - Gap 1 was detected between owners belonging to the general population and owners of pets treated for cancer. This last group had higher expectations in questions regarding assurance. It is of great relevance for these clients to be able to trust their veterinarian and to feel safe when leaving their pet at a veterinary hospital. Furthermore, these clients believe hospital personnel should constantly act in a polite and respectful manner. Similarly, the veterinarian should present sufficient medical knowledge to answer any question they might have about their pet’s health condition.

Owners of pets with cancer recognized Tangible as the least important dimension. In other words, the general appearance of facilities and staff is apparently less relevant than other features englobing SERVQUAL. This dimension was also less considered by the GG even though there was a statistical tendency to score higher. The lowest rank position in the GG is attributed to the responsiveness dimension, where a tendency for lower scores was registered in comparison with the OG. When compared with other services industries both group owners assigned lower importance to the reliability dimension which usually ranks first in many other service industries including health care [26].

These results coincide partially with information found in other publications stating what clients expect from veterinary professionals; they anticipate kindness, sympathy and their concerns to be heard and addressed [16,32,33] all of which can be accounted inside the Empathy dimension of SERVQUAL, the highest rated dimension for the OG. Feeling respected as well as being able to communicate openly with the professional has also acquired enormous importance for owners [33] which are features that can be pertained in the Assurance dimension, another dimension highly classified by owners of pets with cancer. A big issue is that many of the studies made in this field employed questionnaires other than the one provided by the SERVQUAL methodology, making the results very difficult to compare among distinct research papers. Nevertheless, it is obvious that addressing client expectations can be very beneficial. A client with met expectations is a satisfied client, less prone to switching professionals [34,35] and more willing to follow recommendations [34]. Fulfilled client expectations increase satisfaction and compliance, decreases client turnover and also reduces the number of complaints and malpractice claims [32], for it is believed that the majority of complaints in veterinary medicine are due to poor communication between veterinarian and customer, inadequate information provided by the professional [36] and impolite behaviors while approaching the client [32]. Resuming, meeting the customers’ expectations is undoubtedly advantageous for every party involved.

Interestingly owners from the OG perceived veterinary services differently despite being attended by the same professionals and in the same facilities - Gap 2 scoring equally or higher in every dimension and statistically higher in responsiveness and empathy. Although negative emotions such as fear, distress are usually associated with a poorer perception of the service [37], in our study, the OG which would probably be associated with those negative emotions actually scored higher in perceptions of the service. These difference could be attributed to the nature of the treatments: Repeated visits and interactions associated with more intensive schedule follow-up in the OG tend to increase client assessment of the service [37,38]. One other possible explanation was that hospital staff could have been more empathetic with members of OG actually providing the best service to these clients than to members of GG explaining the statistical significant differences in the responsiveness and empathy dimensions which are highly dependent on the performance of veterinary staff.

Although OG members are perceived of having higher perceptions of service quality theoretically leading to increased satisfaction (Q=P−E) that was not the case observed. In fact two dimensions, reliability and empathy - Revealed negative scores on the OG translated on needs not being fulfilled by the veterinary team. It seems that, although generally speaking, members of OG had higher perceptions of value individually, those were not enough to compensate individually higher expectations - Gap 3. This trend had also been observed in the human field where general practice patients (vs. hospital patients), patients with little or no anxiety and patients with disease not affecting quality of life-attributes somehow similar to GG - were more likely to have expectations met [39,40]. Accordingly managers should address more resources to questions such as delivering a service on time and provide accurate clinical and non-clinical information to owners, and veterinary professionals should invest more in developing bedside manner and emotional intelligence skills to increase empathy with their clients.

The final gap noted - Gap 4 consisted in the difference observed between client expectations of what they considered a quality veterinary service and what veterinary professionals consider a quality rich service. This last group clearly overrated tangible aspects of the service when compared to OG and also overrated assurance dimension when compared to GG. This imbalance is well described in the human field [41-43] may lead for healthcare professionals to invest resources in areas nonsolicited by their clients which could in turn be redirected to unmet areas described in Gap 3. Interestingly veterinary professionals showed the same tendency as human doctors to overvalue tangible aspects of services although they differ by not underestimating the other four dimensions [26].

## Conclusion

Owners of pets with oncologic disease constitute a subgroup with particular specificities. Although there has been a notorious lack of research into the singular prospects of oncologic pet owners and veterinary clients overall, the studies that do exist conclude that the fulfillment of client’s expectations is incredibly advantageous. Therefore, it is of the utter importance that veterinarians adapt and improve their capacities to meet their customer’s expectations, this way contributing to the well-being of the veterinary practice.

Hopefully, this study will help to understand and fulfill the special needs of owners of pets with cancer helping to establish a better relation between veterinarian and owner, thus increasing compliance and clinical results while decreasing dissatisfaction and frustration of all parties caused by unmet and unaligned expectations.

## Authors’ Contributions

HG, PS and FLQ planned, researched, designed and edited the manuscript. HG conducted data collection and PS performed data analysis. Data interpretation was conducted by HG, PS, and FLQ. Finally, critical revision was done by IP and JP. All authors read and approved the final manuscript.
